# Measurement bias detection through Bayesian factor analysis

**DOI:** 10.3389/fpsyg.2014.01087

**Published:** 2014-09-29

**Authors:** M. T. Barendse, C. J. Albers, F. J. Oort, M. E. Timmerman

**Affiliations:** ^1^Psychometrics and Statistics, Heymans Institute for Psychological Research, University of GroningenGroningen, Netherlands; ^2^Department of Education, Research Institute of Child Development and Education, University of AmsterdamAmsterdam, Netherlands

**Keywords:** Bayesian structural equation modeling, measurement invariance, uniform bias, nonuniform bias, interaction effects

## Abstract

Measurement bias has been defined as a violation of measurement invariance. Potential violators—variables that possibly violate measurement invariance—can be investigated through restricted factor analysis (RFA). The purpose of the present paper is to investigate a Bayesian approach to estimate RFA models with interaction effects, in order to detect uniform and nonuniform measurement bias. Because modeling nonuniform bias requires an interaction term, it is more complicated than modeling uniform bias. The Bayesian approach seems especially suited for such complex models. In a simulation study we vary the type of bias (uniform, nonuniform), the type of violator (observed continuous, observed dichotomous, latent continuous), and the correlation between the trait and the violator (0.0, 0.5). For each condition, 100 sets of data are generated and analyzed. We examine the accuracy of the parameter estimates and the performance of two bias detection procedures, based on the DIC fit statistic, in Bayesian RFA. Results show that the accuracy of the estimated parameters is satisfactory. Bias detection rates are high in all conditions with an observed violator, and still satisfactory in all other conditions.

## 1. Introduction

Measurement bias research examines whether different respondents show differences in response behavior to test items. In the presence of measurement bias, systematic differences between observed test scores do not validly represent differences in the trait(s) that the test is supposed to measure. Measurement bias is formally defined as a violation of measurement invariance (Oort, 1992, after Mellenbergh, 1989). A test is measurement invariant with respect to *V*, if the following conditional independence holds:

(1) $$f_{1}(X|T=t,\;V=v)=f_{2}(X|T=t),$$

where *X* is a set of observed variables, *T* the trait(s) of interest measured by *X*, and *V* a set of variable(s) other than *T*, which possibly violates measurement invariance; function *f*_1_ is the conditional distribution of *X* given values of *t* and *v*, and *f*_2_ is the conditional distribution of *X* given *t*. If conditional independence does not hold (i.e., *f*_1_ ≠ *f*_2_), the measurement of *T* by *X* is said to be biased with respect to *V*. This is a general definition of measurement bias in the sense that *T* and *V* may be measured on any measurement level (i.e., nominal, ordinal, interval, or ratio), and their mutual relationships may be linear or non-linear. In addition, the violator *V* may be observed or latent.

Structural equation modeling (SEM) offers a flexible framework to test for measurement bias. If the violator is an observed discrete variable, e.g., indicating group membership, measurement bias is typically investigated through multigroup factor analysis (MGFA; Meredith, 1993). In MGFA, differences across groups in intercepts indicate uniform bias (i.e., the size of bias is constant across the trait levels) and differences across groups in factor loadings indicate nonuniform bias (i.e., the size of bias varies with the trait levels). Because MGFA requires an observed discrete violator, its use is rather restricted. In the case of a continuous violator, MGFA is sometimes applied using the categorized version of the violator. However, this practice is to be discouraged, because of the known negative consequences of categorizing variables (e.g., MacCallum et al., 2002; Barendse et al., 2012), and because an attractive alternative is available. This very generally applicable alternative is restricted factor analysis (RFA; Oort, 1992, 1998). The advantages of RFA over MGFA are that RFA can assess measurement bias with respect to any kind of violator (i.e., continuous or discrete, observed, or latent) and with respect to multiple violators simultaneously.

In the linear model associated with RFA, the model for *x*_*i*_, a vector with the observed scores for subject *i* on *J* variables *X*, with a single violator and a single latent trait is defined as

(2) $$x_{i}=u+at_{i}+bv_{i}+ct_{i}v_{i}+de_{i},$$

where *u* is a vector of intercepts for the *J* observed variables, *t*_*i*_ and *v*_*i*_ are the scores of subject *i* on the latent trait *T* and the potential violator *V*, respectively, *e*_*i*_ is a vector of subject *i*'s scores on the standard residual factors *E*, and *a*, *b*, *c*, and *d* are vectors of regression coefficients; the elements of *a* and *d*^2^ are typically denoted as the loadings and residual variances, respectively, and *b* and *c* express possible bias. In case *V* is a categorical variable, dummy variables are used for *V*. If the relationships between the potential violator and the observed variables are entirely explained by the indirect relationships through the latent trait, then the observed variables are unbiased with respect to the possible violator. A non-zero element in *b* indicates uniform bias, and a non-zero element in *c* indicates nonuniform bias. Uniform bias can thus be investigated by testing the direct effects of a violator on the observed variables (Oort, 1992, 1998). Non-uniform bias can be investigated by testing the direct effect of the product of the latent trait and the violator on the observed variables (see Barendse et al., 2010, 2012), either by using latent moderated structures (Klein and Moosbrugger, 2000) or by using a random slope parametrization (Muthén and Asparouhov, 2003).

The RFA method is similar to the multiple indicator multiple cause (MIMIC) method as described by Muthén (1989). They differ in that in the MIMIC model the violator has a causal effect on the latent trait, whereas in RFA the two are correlated.

So far, the vast majority of the literature on testing measurement bias concerns frequentist methods. Alternatively, a Bayesian approach could be used, thereby offering the general advantage that prior knowledge can be incorporated in the analysis. Recently, the first steps were taken toward a Bayesian approach in this context. A Bayesian MGA has been shown to properly detect bias (Lee, 2007). Further, Muthén and Asparouhov (2012) motivate that the Bayesian approach is more suitable to reflect substantive theories, because it allows for an approximate parameter specification, rather than an exact one. As Muthén and Asparouhov (2013) show, a Bayesian MGFA thus allows for approximate measurement invariance testing. Because a Bayesian MGFA is still restricted to cases with a single observed discrete violator, we consider a Bayesian RFA method here. This method is appealing, because it shares the general advantages of a Bayesian approach, while being applicable to assess measurement bias with respect to multiple violators simultaneously, and of any kind (i.e., continuous or discrete, observed or latent).

The purpose of the present paper is to examine the performance of the Bayesian approach to estimate RFA models with interaction effects, in order to detect uniform and nonuniform measurement bias. An additional advantage of Bayesian RFA is that it handles the estimation of the interaction term easier than frequentist (maximum likelihood) RFA. In a simulation study, we will examine the accuracy of the parameter estimates in the Bayesian RFA, and we will compare the performance of two bias detection procedures.

## 2. Methods

Measurement bias in simulated data will be investigated with a Bayesian version of the RFA method. In the data generation, we vary the type of bias (none, only uniform, only nonuniform, both uniform and nonuniform), the type of the continuous violator (observed, latent), and the correlation between the trait and the violator (ρ(*T*,*V*) = 0.0, 0.5). In a fully crossed design with 100 replications for each condition, this yields 4 × 2 × 2 × 100 = 1600 simulated datasets. We additionally introduce a dichotomized violator by performing a median split on the observed continuous violator, and thus analyze 2400 datasets in total. Each data set is analyzed using two different bias detection procedures (to be explained in Section 2.4). The accuracy and efficiency of the parameter estimates is assessed. The performance of the two bias detection procedures is evaluated by examining the proportions of true and false positives.

### 2.1. Data generation

Each data set consists of the observed scores of 500 subjects on 6 items with continuous response scales, and is generated according to the linear model in Equation 2. We draw subject scores *t*, *v*, and *e* from a multivariate standard normal distribution with an identity covariance matrix in the condition with ρ = 0.0; in the condition with ρ = 0.5, the element in the covariance matrix associated with *t* and *v* is set to 0.500. We have chosen the value of ρ = 0.0 as we presume that an absence of linear dependency is the easiest condition in this respect; further we have chosen the value of ρ = 0.5, as its corresponds to a “large correlation,” according to Cohen's rules of thumb (Cohen, 1988). The intercepts *u* are set at zero, and the regression coefficients *a* and *d* are set at 1.000, for all items. Bias is introduced in the first item only, in such a way that the amount of bias is in line with other bias detection studies (e.g., Oort, 1998). That is, we set parameter *b* = 0.400 to obtain uniform bias and parameter *c* = 0.400 to obtain nonuniform bias—the remaining elements of *b* and *c* are fixed at zero. Table 1 gives an overview of the chosen parameter values for the first item. With these values, if *T* and *V* are uncorrelated, the expected percentage of total observed item variance due to the bias is approximately 7% in conditions with only uniform or nonuniform bias and approximately 14% in conditions with both uniform and nonuniform bias. If *T* and *V* are correlated, these percentages are 6% (in case of uniform bias), 7% (nonuniform bias), and 13% (both uniform and nonuniform bias).

**Table 1:** **Parameter values for 4 (type of bias) × 2 (correlation between trait and violator) = 8 data generation conditions**.

	**Unstandardized values of Item 1 parameters**				
	***a***	***b***	***c***	***d***	**σ^2^(*X*)**
**ρ(*T,V*) = 0.0**					
No bias	1.000	0.000	0.000	1.000	2.000
Uniform	1.000	0.400	0.000	1.000	2.160
Nonuniform	1.000	0.000	0.400	1.000	2.160
Both	1.000	0.400	0.400	1.000	2.320
**ρ(*T,V*) = 0.5**					
No bias	1.000	0.000	0.000	1.000	2.000
Uniform	1.000	0.400	0.000	1.000	2.560
Nonuniform	1.000	0.000	0.400	1.000	2.200
Both	1.000	0.400	0.400	1.000	2.760

u = 0, μ(T) = μ(V) = μ(E) = 0, σ^2^(T) = σ^2^(V) = σ^2^(E) = 1; All values pertain to the parameters of Item 1, which is biased in all conditions with bias; parameters of all other items have a = 1, b = 0, c = 0, and d = 1 in all conditions. See Appendix 1 in Supplementary Material for the computation of σ^2^(X).

The violator can either be a continuous latent, a continuous observed or a dichotomous observed variable. In conditions with a continuous latent violator, we introduce three observed variables indicative of the latent violator, which follow a linear factor model. We draw the scores on the latent violator and the residuals independently from a standard normal distribution, and use factor loadings equal to one. In conditions with a continuous observed violator, we draw *V* from a standard normal distribution. In conditions with a dichotomous observed violator, we perform a median split on the continuous observed violator and conveniently choose *V* = −1 for one group and *V* = 1 for the other group to model the interaction effects.

### 2.2. Bayesian structural equation modeling and bias detection

Bayesian SEM to detect bias is embedded in Bayesian theory and the associated computational procedures. Bayesian theory combines prior information about the distributions of parameters (called the prior distributions) and the distributions of the data under any SEM model (*M*). Let θ denote a vector of unknown parameters that are considered to be random. As the observed data and the parameters are random, we model the joint probability (called the posterior distribution) as a function of the conditional distribution of the data given the parameters *p*(*X*|θ, *M*) and the prior distribution of the parameters *p*(θ). More formally this is defined in Bayes' rule:

(3) $$p(X,\theta |M)=\frac{p(X|\theta ,M)p(\theta )}{p(X)},$$

where *p*(*X*) normalizes the conditional distribution. As normalizing does not involve any model parameters, Equation 3 can be rewritten as

(4) p(X,θ|M)∝p(X|θ,M)p(θ).

Equations 3 and 4 show that the posterior density function includes sample information and prior information. If the prior distribution of θ is so-called uninformative, the posterior density function is proportional to the log-likelihood function. Ideally, a closed form solution of the posterior can be obtained via integration. In practice, one simulates a sufficiently large number of observations from the posterior distribution with Markov Chain Monte Carlo sampling to approximate statistics such as the mean or mode of parameters. Tanner and Wong (1987) introduced the idea to analyze latent variables in a Bayesian context, which is particularly useful for SEM. Latent variables are then treated as hypothetical missing data and the posterior distribution is analyzed on the basis of the complete data.

### 2.3. Bayesian model selection

In Bayesian bias detection we aim at identifying the biased item(s) and the nature of the bias. We therefore compare competing models (i.e., models with and without parameters to account for bias) and select the best fitting model using the deviance information criterion (DIC; see Spiegelhalter et al., 2002). The DIC is a measure of model fit that penalizes for complexity. Under a competing model *M*_*k*_, the DIC is defined as

(5) $$DIC_{k}=-\frac{2}{L}\sum_{l\;=\;1}^{L}\log p(Y|\theta_{k}^{\left(l\right)},M_{k})+2d_{k}$$

where θ_*k*_ is a vector of unknown parameters of dimension *d*_*k*_, and {θ^(*l*)^: *l* = 1, …, *L*} is a sample of observations simulated from the posterior distribution. The model with the smallest DIC has the highest chance to predict a replicate data set.

Lee (2007) already concluded that a very small difference in DIC values of competing models could be misleading. Also, Lunn et al. (2009) outlines a variety of reasons that could distort the DIC values. We therefore compare our reference model—to be defined later—with competing models and apply two different cut-off values, namely a strict cut-off and a liberal cut-off, to be defined later.

### 2.4. Measurement bias detection

**Figure 1:**
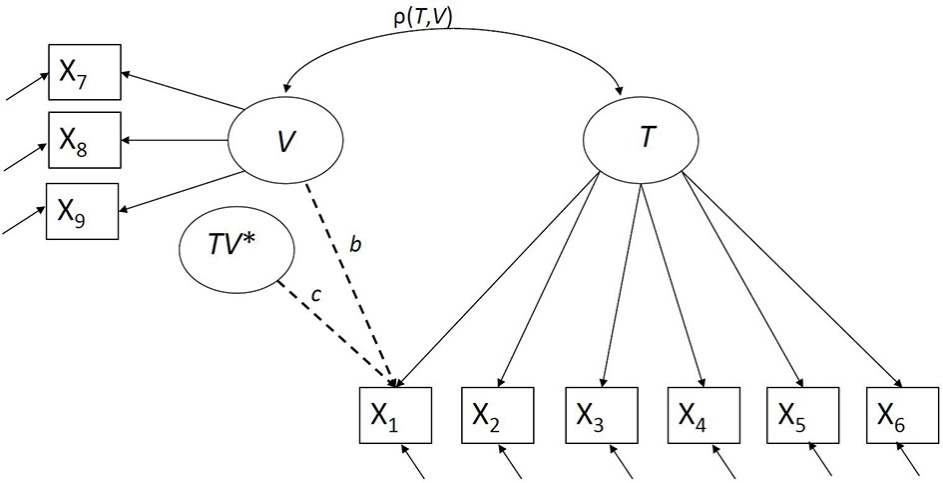
Bias detection with respect to a continuous latent violator *V*, TV^*^ indicates the interaction between *T* and *V*.

In a model accounting for bias, we include a direct effect of the violator on the item score to account for uniform bias and a direct effect of the product of the trait and the violator on the item score to account for nonuniform bias. We consider three types of violators (latent continuous, observed continuous, and observed dichotomous), and therefore define three related Bayesian RFA models to model both uniform and nonuniform bias. A bias detection model with respect to a continuous latent violator is graphically displayed in Figure 1. To evaluate the approach, we will examine the accuracy and efficiency of the parameter estimates, and the performance in detecting bias with two bias detection procedures.

#### 2.4.1. Parameter estimates

To evaluate the accuracy and the efficiency of the parameter estimates expressing uniform and nonuniform bias, we estimate for each simulated data set the model according to Equation 2; herewith, we fix the elements of *b* and *c* associated with items 2–6 (which are non-biased) at zero. Of each converged estimated model, we consider for the first item the posterior distribution of *b* (indicating uniform bias) and the posterior distribution of *c* (indicating nonuniform bias). For each posterior distribution, we compute its mean (denoted as θ_*b*_ for *b* and θ_*c*_ for *c*) and the Monte Carlo standard error (denoted as se(θ_*b*_) and se(θ_*c*_), respectively) using the time series standard error as implemented in CODA (Roberts, 1996; Plummer et al., 2006).

As the accuracy measure, we compute for each condition the estimation bias, as the average of the means of the posterior distribution minus the chosen population values [i.e., (m(θ_*b*_) − *b*) and (m(θ_*c*_) − *c*)]. (Throughout this paper, we shall use the notation m(·) to denote a mean.) As the efficiency measure, we compute for each condition the standard deviation of the means of the posterior distribution (i.e., sd(θ_*b*_) and sd(θ_*c*_)). To evaluate the Monte Carlo sampling accuracy, we compute the mean of the time-series standard errors across the replicates [i.e., m(se(θ_*b*_)) and m(se(θ_*c*_))].

#### 2.4.2. Two procedures to detect bias

In the *single run procedure*, we consider for each of the *j* (*j* = 1, …, 6) items indicative of the trait, a reference model and compare that model to five ((*j*′ = 1, …, 6), *j*′ ≠ *j*) competing models. Both the reference and competing models have parameters accounting for both uniform and nonuniform bias. For each item *j*, we consider the model with bias parameters for that item as the reference model. We compare each reference model to each of the five competing models ((*j*′ = 1, …, 6), *j*′ ≠ *j*), by considering each of five DIC differences, as the DIC value of the reference model minus the DIC value of the competing models. With the strict cut-off value, the item with bias parameters in the reference model is indicated as biased if the associated DIC difference is negative. With the liberal cut-off value, the DIC difference should be smaller than −10, to flag item *j* as biased. The value of 10 was chosen, as it is thought to reflect a substantial difference in model fit (MRC Biostatistics Unit, 2006).

In the *model difference procedure*, we consider for each of the *j* (*j* = 1, …, 6) items indicative of the trait, a reference model with bias parameters for item *j*, and compare that to a nested model, namely without any bias parameters. We apply a strict cut-off value, considering item *j* to be biased whenever the DIC of the reference model is lower than that of the nested model. With the liberal cut-off value, item *j* is indicated as biased when the value of the DIC of the reference model of item *j* is at least 10 lower than that of the nested model.

For each of the two procedures, we calculate proportions of true and false positives. A true positive is a biased item that is correctly detected; a false positive is an unbiased item that is incorrectly detected as biased. Proportions of true positives and false positives are interpreted similar to power and Type I error, respectively.

### 2.5. Analysis

The Bayesian RFA was implemented in R (version 3.02; R Core Team, 2013), using the packages R2OpenBUGS, BRugs, CODA (Plummer et al., 2006) and in BUGS (version 3.2.2; Lunn et al., 2009). All models are fitted to raw data. To estimate non-linear effects, we employ the approach described by Lee (2007), which partitions the latent variables into a linear and a non-linear part with appropriate identification conditions. BUGS uses the Gibbs sampler and the Metropolis-Hastings algorithm for efficient estimation of the Bayesian RFA.

Measurement bias is detected with respect to a continuous latent, a continuous observed and a dichotomous observed violator. Where possible, we use conjugate priors. Conjugate priors are such that the posterior distribution is of the same family as the prior, usually lowering the computational demands of the algorithms drastically. The conjugate priors that we use for each of these models are the normal (for the unknown mean), gamma (for the variance), and the inverse Wishart (for the correlation between latent variables) distributions, as these distributions lead to good results in Bayesian SEM (Lee, 2007; Lee and Song, 2012). We use informative priors that are based on the chosen population values (see Table 1). The software cannot work with an observed violator directly. As a workaround, we introduce the violator *V* as a latent variable with a variance very close to zero, thus making it “practically observed.” In conditions with a dichotomous observed violator, we use a prior with a strongly peaked hyperprior for the variance of the violator. Details about the prior elicitation are provided in Appendix 2 in Supplementary Material. All scripts used in this paper are available from http://www.casperalbers.nl.

The number of iterations has been decided upon using the Raftery and Lewis diagnostic (Raftery and Lewis, 1992, 1995). We used 4000 iterations for the burn-in phase and 8000 iterations for the model estimation. For models with a latent violator we used 21000 iterations for the model estimation, as convergence appeared to be slower in these instances in a small pilot study. For each model, we simulated three chains with different initial values for each of the parameters. To decide on the convergence we inspect the Gelman and Rubin's convergence diagnostic (Gelman and Rubin, 1992). This diagnostic compares the within-chain and between-chain variance and a value above 1.1 is an indication of lack of convergence (Gelman et al., 2013). Additionally, we inspected the Geweke convergence diagnostic (Geweke, 1992), which is based on a test for equality of first and the last part of a single Markov chain with the difference between sample means devided by the standard errors expressed in Z-scores.

In addition to the analysis described, we also perform a sensitivity analysis in conditions with a correlated trait and violator (i.e., ρ(*T*,*V*) = 0.5), and both uniform and nonuniform bias (i.e., Conditions 8, 16, and 24, see Table 2), to examine to what extent our choice of the priors influences the parameter estimates. To study the impact of the prior inputs in the Bayesian method, we consider the priors associated with parameters in the absence of bias as inaccurate priors for the bias parameters.

**Table 2:** **Accuracy and efficiency in the Bayesian RFA**.

	**Bias**	**Cond.**	**Conv.**	***b***			***c***		
				**m(θ_*b*_) − *b***	**sd(θ_*b*_)**	**m(se(θ_*b*_))**	**m(θ_*c*_) − *c***	**sd(θ_*c*_)**	**m(se(θ_*c*_))**
**CONTINUOUS LATENT VIOLATOR**									
ρ(*T*,*V*) = 0.0	No bias	1	0.95	0.010	0.066	0.001	−0.005	0.073	0.001
	Uniform	2	0.59	0.009	0.062	0.001	0.000	0.078	0.001
	Nonuniform	3	0.93	0.007	0.065	0.001	0.035	0.079	0.001
	Both	4	0.65	0.024	0.065	0.001	0.031	0.087	0.001
ρ(*T*,*V*) = 0.5	No bias	5	0.97	−0.011	0.081	0.001	−0.004	0.059	0.001
	Uniform	6	0.93	0.005	0.082	0.001	0.000	0.059	0.001
	Nonuniform	7	0.96	−0.014	0.083	0.001	0.021	0.072	0.001
	Both	8	0.92	0.002	0.083	0.001	0.022	0.070	0.001
**CONTINUOUS OBSERVED VIOLATOR**									
ρ(*T*,*V*) = 0.0	No bias	9	1.00	0.006	0.054	0.001	−0.002	0.054	0.001
	Uniform	10	0.91	−0.003	0.047	0.001	0.005	0.057	0.001
	Nonuniform	11	0.92	0.000	0.056	0.001	0.008	0.057	0.001
	Both	12	0.92	−0.008	0.059	0.001	0.015	0.051	0.001
ρ(*T*,*V*) = 0.5	No bias	13	0.80	−0.008	0.054	0.001	−0.006	0.047	0.001
	Uniform	14	0.55	−0.006	0.068	0.001	0.008	0.053	0.001
	Nonuniform	15	0.75	−0.002	0.068	0.001	0.013	0.055	0.001
	Both	16	0.52	−0.004	0.063	0.001	0.006	0.046	0.001
**DICHOTOMIZED OBSERVED VIOLATOR (AFTER MEDIAN SPLIT OF THE CONTINUOUS OBSERVED VIOLATOR)**									
ρ(*T*,*V*) = 0.0	No bias	17	1.00	−0.002	0.041	0.001	−0.004	0.054	0.001
	Uniform	18	0.99	−0.089	0.046	0.001	0.003	0.055	0.001
	Nonuniform	19	1.00	0.004	0.051	0.001	−0.071	0.063	0.001
	Both	20	0.98	0.087	0.063	0.001	−0.062	0.058	0.001
ρ(*T*,*V*) = 0.5	No bias	21	0.94	−0.009	0.051	0.001	0.006	0.056	0.001
	Uniform	22	0.71	−0.112	0.056	0.001	−0.002	0.051	0.001
	Nonuniform	23	0.93	−0.004	0.055	0.001	−0.022	0.071	0.001
	Both	24	0.68	−0.131	0.054	0.001	0.021	0.067	0.001

Cond., Condition; Conv., proportion of converged solutions (of 100 replicates); All summary measures of the parameter estimates are calculated over the converged solutions only.

## 3. Results

After applying the Bayesian RFA to each of the 2400 data sets, we find that the algorithm does not always converge, as indicated by a value exceeding one on Gelman and Rubin's convergence diagnostic (Gelman and Rubin, 1992). Geweke's convergence diagnostic (Geweke, 1992) is much more conservative: it has values larger than the standard threshold value of 2 for at least one chain (out of three) in the vast majority of the simulated data sets, in all conditions. We therefore report convergence according to the Gelman-Rubin diagnostic.

As shown in Tables 2–4, we encounter convergence problems especially in conditions that contain uniform bias and a latent violator without a correlation between the trait and the violator and in conditions with an observed violator with a correlation between the trait and the violator.

Non-convergence results are not further analyzed and ignored when assessing the parameter estimates and detecting bias.

### 3.1. Parameter estimates

Table 2 gives the measures of accuracy and efficiency of the estimated parameters that are associated with the parameters that express uniform (i.e., parameter *b*) and nonuniform (i.e., parameter *c*) bias in the first item: the estimation bias (i.e., (m(θ_*b*_) − *b*) and (m(θ_*c*_) − *c*), the efficiency (i.e., sd(θ_*b*_) and sd(θ_*c*_)), and the Monte Carlo accuracy (i.e., m(se(θ_*b*_)) and m(se(θ_*c*_))).

As can be seen in Table 2, the estimation bias appears rather low in the conditions with a continuous latent violator, both for the parameter expressing uniform bias (i.e., *b*), and nonuniform bias (i.e., *c*), with a maximum observed estimation bias across all conditions of 0.035. The conditions with a continuous observed violator show a similar pattern, with the largest estimation bias being 0.015.

In the conditions with a dichotomized observed violator, we observe relatively large estimation bias for, firstly, the parameter expressing uniform bias in those conditions that include uniform bias (with a maximum absolute estimation bias of 0.131) and, secondly, but to a lesser extent, the parameter expressing nonuniform bias in those conditions that include nonuniform bias (with a maximum absolute estimation bias of 0.071). With a dichotomized violator, the parameters that represent bias are underestimated.

Across all conditions, the efficiency of the parameters related to uniform and nonuniform bias in the Bayesian RFA is reasonably good, as indicated by the small values of the efficiency parameters (sd(θ_*b*_) and sd(θ_*c*_)) (ranging from from 0.041 to 0.087). We further note that the means of time-series standard errors are small. We therefore conclude that the Monte Carlo accuracy is high.

#### 3.1.1. Single run procedure to detect bias

Table 3 gives the single run procedure results; the convergence rates, the quantile (i.e., 5, 50, and 95) values of the DIC difference between the reference model and the competing model with the most deviating DIC value, and the proportions of true positives and false positives at the strict and the liberal DIC cut-off values. The convergence rates in the single run procedure show considerable variability across conditions (ranging from 0.07 to 0.99), with particular low values for the models with a continuous latent violator with a substantially correlated latent trait and violator.

**Table 3:** **Bias detection with the single run procedure**.

		**Cond.**	**Conv.**	**Biased items^a^**					**Unbiased items^b^**				
				**Δ DIC**			**TP**		**Δ DIC**			**FP**	
				***Q*_05_**	***Q*_50_**	***Q*_95_**	**Strict**	**Liberal**	***Q*_05_**	***Q*_50_**	***Q*_95_**	**Strict**	**Liberal**
**CONTINUOUS LATENT VIOLATOR**													
ρ(*T*,*V*) = 0.0	No bias	1	0.73	–	–	–	–	–	−10	0	0	0.304	0.014
	Uniform	2	0.45	−100	−70	−42	1.000	1.000	−20	0	0	0.387	0.093
	Nonuniform	3	0.67	−110	−70	−40	1.000	1.000	−20	0	0	0.382	0.063
	Both	4	0.52	−185	−125	−86	1.000	1.000	−20	0	0	0.423	0.096
ρ(*T*,*V*) = 0.5	No bias	5	0.75	–	–	–	–	–	−10	0	0	0.282	0.020
	Uniform	6	0.74	−80	−50	−27	1.000	1.000	−20	0	0	0.432	0.081
	Nonuniform	7	0.78	−140	−85	−40	1.000	1.000	−20	0	0	0.426	0.079
	Both	8	0.75	−193	−130	−70	1.000	1.000	−20	−10	0	0.528	0.157
**CONTINUOUS OBSERVED VIOLATOR**													
ρ(*T*,*V*) = 0.0	No bias	9	0.96	–	–	–	–	–	−8	−2	0	0.776	0.016
	Uniform	10	0.88	−103	−71	−50	1.000	1.000	−13	−3	0	0.748	0.093
	Nonuniform	11	0.89	−110	−71	−44	1.000	1.000	−12	−3	0	0.742	0.079
	Both	12	0.88	−175	−138	−93	1.000	1.000	−14	−4	0	0.764	0.143
ρ(*T*,*V*) = 0.5	No bias	13	0.28	–	–	–	–	–	−8	−2	0	0.756	0.030
	Uniform	14	0.07	−68	−52	−34	1.000	1.000	−14	−2	0	0.657	0.171
	Nonuniform	15	0.18	−106	−77	−46	1.000	1.000	−12	−3	0	0.722	0.100
	Both	16	0.08	−147	−124	−96	1.000	1.000	−18	−7	0	0.750	0.300
**DICHOTOMIZED OBSERVED VIOLATOR**													
ρ(*T*,*V*) = 0.0	No bias	17	0.99	–	–	–	–	–	−9	−2	0	0.791	0.022
	Uniform	18	0.99	−70	−45	−28	1.000	1.000	−12	−2	0	0.739	0.089
	Nonuniform	19	0.99	−73	−45	−22	1.000	1.000	−11	−3	0	0.737	0.057
	Both	20	0.96	−119	−88	−55	1.000	1.000	−13	−3	0	0.760	0.104
ρ(*T*,*V*) = 0.5	No bias	21	0.69	–	–	–	–	–	−8	−2	0	0.775	0.017
	Uniform	22	0.38	−56	−28	−14	1.000	0.974	−11	−3	0	0.758	0.058
	Nonuniform	23	0.62	−78	−50	26	1.000	1.000	−11	−3	0	0.745	0.055
	Both	24	0.32	−118	−84	−54	1.000	1.000	−16	−4	0	0.756	0.131

Cond., Condition; Conv., proportion of converged solutions; Δ DIC denotes the difference in DIC between the reference model and the competing model;

a Quantile DIC difference values (i.e., 05, 50, 95), and proportions of true positives (TP) are calculated over the converged solutions [of 1 (biased item) × 100 (replicates) = 100 solutions];

b Quantile DIC values (i.e., 05, 50, 95), and proportions of false positives (FP) are calculated over the converged solutions, which are 6 (non-biased items) × 100 (replicates) = 600 solutions in Conditions 1, 5, 9, 13, 17 and 21, and 5 (non-biased items) × 100 (replicates) = 500 solutions in all other conditions.

As can be seen in Table 3, the proportions of true positives (i.e., indicating the bias whenever it is present) are 1.000 in all conditions with the strict criterion, and ranges from 0.974 to 1.000 with the liberal criterion. Thus, both criteria are very successful in detecting the bias. The quantiles of the DIC difference values give an indication of the power to identify items with bias. These DIC difference values are highly negative in conditions with both uniform and nonuniform bias, but also substantial in conditions with only uniform or nonuniform bias. In conditions with a dichotomous observed violator, we observe smaller negative DIC difference values, suggesting a lower power.

The proportions of false positives with the strict cut-off value are very high (ranging from 0.304 to 0.791). With a liberal cut-off value, the proportions of false positives were reasonably low (from 0.014, with a maximum of 0.300); they appear somewhat higher in conditions with both uniform and nonuniform bias and a correlated trait and violator. Considering the performance in terms of both true positives and false positives, the liberal cut-off value seems best suited for bias detection with the single run procedure.

#### 3.1.2. Model difference procedure to detect bias

Table 4 shows the results of the model difference procedure: the convergence proportions, the quantile (i.e., 5, 50, and 95) values of the DIC difference between the reference model (i.e., with parameters to represent bias) and the nested model (i.e., without parameters to represent bias), and the proportions of true positives and false positives at the strict and the liberal cut-off values. The convergence rates show considerable variability across conditions (ranging from 0.49 to 1.00). Overall, the convergence rate is higher in the model difference procedure than in the single run procedure, because the former requires only two, and the latter *J* = 6 models to be estimated.

**Table 4:** **Bias detection with the model difference procedure**.

		**Cond.**		**Biased items^a^**					**Unbiased items^b^**					
			**Conv.**	**Δ DIC**			**TP**		**Conv.**	**Δ DIC**			**FP**	
				***Q*_05_**	***Q*_50_**	***Q*_95_**	**Strict**	**Liberal**		***Q*_05_**	***Q*_50_**	***Q*_95_**	**Strict**	**Liberal**
**CONTINUOUS LATENT VIOLATOR**														
ρ(*T*,*V*) = 0.0	No bias	1	–	–	–	–	–	–	0.93	−10	0	10	0.102	0.007
	Uniform	2	0.59	−100	−60	−40	1.000	1.000	0.86	−10	0	10	0.255	0.042
	Nonuniform	3	0.93	−110	−70	−30	1.000	0.989	0.92	−10	0	10	0.219	0.032
	Both	4	0.65	−188	−120	−82	1.000	1.000	0.88	−20	0	10	0.305	0.068
ρ(*T*,*V*) = 0.5	No bias	5	–	–	–	–	–	–	0.94	−10	0	10	0.115	0.012
	Uniform	6	0.93	−80	−40	−20	1.000	0.978	0.93	−10	0	10	0.263	0.045
	Nonuniform	7	0.96	−140	−80	−40	1.000	1.000	0.95	−10	0	10	0.213	0.023
	Both	8	0.92	−190	−120	−70	1.000	1.000	0.94	−20	0	10	0.383	0.097
**CONTINUOUS OBSERVED VIOLATOR**														
ρ(*T*,*V*) = 0.0	No bias	9	–	–	–	–	–	–	0.99	−4	1	5	0.232	0.002
	Uniform	10	0.91	−100	−67	−47	1.000	1.000	0.99	−9	0	5	0.485	0.028
	Nonuniform	11	0.92	−107	−67	−40	1.000	1.000	0.99	−9	0	4	0.451	0.032
	Both	12	0.92	−173	−137	−92	1.000	1.000	0.99	−12	−1	4	0.578	0.073
ρ(*T*,*V*) = 0.5	No bias	13	–	–	–	–	–	–	0.77	−5	2	5	0.274	0.009
	Uniform	14	0.52	−90	−50	−27	1.000	1.000	0.61	−13	−1	4	0.529	0.082
	Nonuniform	15	0.73	−121	−81	−47	1.000	1.000	0.76	−10	0	4	0.479	0.037
	Both	16	0.49	−167	−125	−94	1.000	1.000	0.64	−16	−3	4	0.672	0.172
**DICHOTOMIZED OBSERVED VIOLATOR**														
ρ(*T*,*V*) = 0.0	No bias	17	–	–	–	–	–	–	1.00	−4	1	5	0.239	0.008
	Uniform	18	0.99	−66	−42	−23	1.000	1.000	1.00	−9	0	4	0.390	0.032
	Nonuniform	19	1.00	−71	−43	−20	1.000	0.980	0.99	−7	0	4	0.401	0.028
	Both	20	0.98	−115	−84	−51	1.000	1.000	0.99	−10	0	4	0.462	0.044
ρ(*T*,*V*) = 0.5	No bias	21	–	–	–	–	–	–	0.94	−5	1	5	0.266	0.005
	Uniform	22	0.70	−53	−25	−10	1.000	0.943	0.86	−8	0	4	0.456	0.019
	Nonuniform	23	0.93	−79	−47	−21	1.000	1.000	0.93	−8	1	4	0.392	0.017
	Both	24	0.66	−116	−81	−55	1.000	1.000	0.83	−13	−1	4	0.550	0.099

Cond., Condition; Conv., proportion of converged solutions; Δ DIC denotes the difference in DIC between the reference model and the competing model;

a Quantile DIC difference values (i.e., 05, 50, 95), and proportions of true positives (TP) are calculated over the converged solutions [of 1 (biased item) × 100 (replicates) = 100 solutions];

b Quantile DIC values (i.e., 05, 50, 95), and proportions of false positives (FP) are calculated over the converged solutions, which are 6 (non-biased items) × 100 (replicates) = 600 solutions in Conditions 1, 5, 9, 13, 17, and 21, and 5 (non-biased items) × 100 (replicates) = 500 solutions in all other conditions.

As can be seen in Table 4, the proportions of true positives (i.e., indicating bias when it is present) are very high in all conditions; both using the strict criterion (all 1.000) as using the liberal criterion (ranging from 0.943 to 1.000).

The quantiles of the DIC difference values give an indication of the power to identify items with bias. These DIC difference values are highly negative in conditions with both uniform and nonuniform bias, for all conditions with a continuous violator. In conditions with a dichotomous violator, we observe smaller negative DIC difference values, suggesting a lower power.

In all conditions with a continuous violator, the proportions of false positives with the strict cut-off value are high (ranging from 0.102 to 0.672), and with the liberal cut-off value reasonably low (maximally 0.172). For the dichotomized violator a similar pattern is observed. When applying the model difference procedure, the liberal cut-off value appears to perform better than the strict cut-off value, in terms of a proper balance between true positives and false negatives.

#### 3.1.3. Sensitivity analyses

To assess the sensitivity to the choice of the priors for those parameters that express uniform and nonuniform bias, we reanalyzed the simulated data sets in the “most difficult” conditions: with both uniform and nonuniform bias and a correlated trait and violator (i.e., Conditions 8, 16, and 24) using clearly incorrect priors. That is, for the parameters expressing the bias, we use priors that reflect an absence of bias (i.e., a normal distribution with a mean of zero, for *b* and *c*). Table 5 shows the measures of accuracy and efficiency of the estimated parameters, in a similar way as reported in Table 2. Comparing the results of Tables 2, 5 shows that, in case of a continuous violator, the estimation bias is still remarkably low when faced with clearly incorrect priors (all absolute values lower than 0.016). Also the efficiency and MCM standard errors and the convergence rates of these two conditions are comparable to those in Table 2. Also in case of a dichotomized violator, the estimation bias (with values −0.130 and 0.016) is very similar to the corresponding values in Table 2. Proportions of true and false positives hardly change when using the clearly incorrect prior, as has been verified (but not reported). We conclude that inadequate priors hardly influence the parameter estimates in all conditions, at least with the iteration length used in this simulation study.

**Table 5:** **Estimation bias of the sensitivity analysis**.

**Violator**	**Cond.**	**Conv.**	**m(θ_*b*_) − *b***	**sd(θ_*b*_)**	**m(se(θ_*b*_))**	**m(θ_*c*_) − *c***	**sd(θ_*c*_)**	**m(se(θ_*c*_))**
Continuous latent	8	0.95	−0.002	0.081	0.001	0.016	0.070	0.001
Continuous observed	16	0.48	0.014	0.058	0.001	0.006	0.058	0.001
Dichtomized observed	24	0.75	−0.130	0.056	0.001	0.016	0.061	0.001

Cond., Condition; Conv., proportion of converged solutions; All summary measures are calculated over the converged solutions only and are using the same notation as in Table 2.

## 4. Discussion

In this article, we consider a Bayesian RFA approach for the detection of uniform and nonuniform bias. Results of a simulation study show that the parameter estimates of this proposed Bayesian RFA are reasonably accurate and efficient. With a dichotomized observed violator we find less accurate results, which is due to a loss of information and a reduction of the effect size. Our results thus support the validity of the well-know criticism on the median split (see, e.g., MacCallum et al., 2002). This suggests that the use of MGFA in cases with a continuous observed violator, with its associated necessity to dichotomize, should be discouraged. We used informative priors to obtain accurate and efficient results for the parameter estimates. Our sensitivity analysis shows that clearly inaccurate priors for the parameters expressing the bias also yield accurate and efficient estimates. This result might be different when working with a smaller sample than the *n* = 500 used in this paper. The smaller the sample size, the larger the influence of the prior distribution. In practice, to obtain priors we have to utilize prior information from different sources available (e.g., knowledge of experts or analyses of similar data), or perform an auxiliary estimation on a part of the data.

Results show that the Bayesian RFA is hindered by convergence problems, particularly in conditions with uniform bias. We used the Raftery and Lewis diagnostic to determine the number of iterations, but noticed in small experiments that doubling the number of iterations still decreased the number of convergence problems, according to the Gelman and Rubin diagnostic and Geweke diagnostics, substantially. For example, for condition 16 in Table 2, doubling the number of iterations increased the convergence rate from 52 to 69%. Thus there are indications that several of the convergence and estimation problems encountered in this simulation study, can be overcome in an empirical context through solutions such as choosing more chains, performing more iterations, and changing the initial values of the chains. Studying convergence properties for a variety of settings, including a variety of sample sizes, would be an interesting topic for future research.

The bias detection rates of both the single run procedure and the model difference procedure, calculated with either a strict or a liberal cut-off value, are very good. In both bias detection procedures, the distribution of the DIC difference values in the various conditions shows that the power to detect bias is the highest in conditions with a continuous observed violator. In conditions with a dichotomous observed violator there is a reduction of power, indicated by lower DIC difference values. In general, nonuniform bias is detected about as well as uniform bias is. However, if we focus on the DIC difference values, conditions with a independent trait and violator and nonuniform bias have smaller DIC difference values than conditions with uniform bias. In conditions with a dependent trait and violator, it is the other way round. This might be related to the fact that both the dependency between the trait and the violator and the bias are positive which may amplify each other.

Overall, the false positive rates are too large with a strict DIC cut-off value. Given the fact that a liberal cut-off value yields satisfactory bias detection results, we recommend a liberal DIC cut-off value (see also Lee, 2007). The false positive rates of the liberal DIC cut-off value are acceptable in all conditions and clearly lower in the model difference procedure. This might be due to a more precise estimation of the DIC difference procedure, as the model difference procedure directly compares a model with and without parameters to assess bias.

As an alternative to a liberal cut-off value, it might be helpful to detect bias in an iterative procedure. In this iterative procedure, the item associated with the largest DIC difference value is considered biased. In a second run, this bias is taken into account by allowing parameters that express bias in the model, and the bias test is conducted on the remaining items. As none of the remaining items is considered biased or half of the items are detected as biased, the iterative procedure stops (see Barendse et al., 2012, for an implementation in the frequentist framework).

Overall, for bias detection with the Bayesian RFA, both procedures with a liberal cut-off value are successful under the conditions studied. The model difference procedure appears to be more powerful in detecting bias and is therefore preferable over the single run procedure. The results presented indicate that the Bayesian RFA method is promising to assess measurement bias. It can be used to assess measurement bias with respect to multiple violators simultaneously, and of any kind (i.e., continuous or discrete, observed or latent).

For further research on the Bayesian RFA, it is useful to investigate model performance under other conditions, including larger numbers of observed items and varying the size of the bias. The size of the bias can be varied both in terms of severity and number of biased items. Additionally, more complicated models, with more than one item with bias, could be investigated. Further, extending the model with a latent categorical violator might be a useful extension. It may also be useful to consider alternative, promising, criteria for bias detection, such as Bayes factors and path sampling which both can deal with non-linearity (Lee, 2007). Finally, it would be highly interesting to see whether to theoretical advantages of Bayesian RFA are of use in empirical practice, by applying the methodology of this paper to empirical data.

## Author contributions

All authors meet the criteria for authorship. All authors contributed substantially to the conception and design of the work, and drafting and finalizing the paper. M. T. Barendse, C. J. Albers, and M. E. Timmerman designed the simulation study, and M. T. Barendse and C. J. Albers programmed the simulation study.

### Conflict of interest statement

The authors declare that the research was conducted in the absence of any commercial or financial relationships that could be construed as a potential conflict of interest.
