# Supplementary material for: Measurement bias detection through Bayesian factor analysis
Source: Front Psychol. 2014 Sep 29;5:1087. doi: 10.3389/fpsyg.2014.01087 (PMC4212259; doi:10.3389/fpsyg.2014.01087)
Supplement: Supplementary file 1 [file Presentation1.PDF]

# APPENDICES

## 1 COMPUTATION OF $\sigma^2$ IN TABLE 1

The computation of $\mathrm{Var}(X) = \sigma^2(X)$ is done via the formula

$$\sigma^2(X) = a^2 + b^2 + c^2(1+\rho^2) + d^2 + 2ab\rho.$$

In this appendix we outline how to arrive at this formula. For simplicity, we write $X$ for $X_1$.

Consider the Structural Equation Model in Figure 1 where $T$, $V$ are both standard normally distributed with covariance $\rho$ and product $T \times V = TV^*$. Apart from $T$, $V$, $TV^*$ we have a loading $d$ of another standard normal variable on $X$, say $W$ (denoted by the arrow at the bottom of the figure). The loadings on $X$ by $T$, $V$ and $TV^*$ are denoted by $a$, $b$ and $c$, respectively. Basic probability dictates that

$$\begin{aligned} \mathrm{Var}(X) &= a^2\mathrm{Var}(T) + b^2\mathrm{Var}(V) + c^2\mathrm{Var}(TV^*) + d^2\mathrm{Var}(W) \\ &+ 2ab\mathrm{Cov}(T,V) + 2ac\mathrm{Cov}(T,TV^*) + 2bc\mathrm{Cov}(V,TV^*). \end{aligned}$$

Since $W$ is uncorrelated with $T$, $V$ and $TV^*$, its covariances are eliminated from above formula. Subsequently, we fill in $\mathrm{Var}(T) = \mathrm{Var}(V) = \mathrm{Var}(W) = 1$ and $\mathrm{Cov}(T,V) = \rho$ to obtain

$$\begin{aligned} \mathrm{Var}(X) &= a^2 + b^2 + c^2\mathrm{Var}(TV^*) + d^2 + 2ab\rho + 2ac\mathrm{Cov}(T,TV^*) + 2bc\mathrm{Cov}(V,TV^*) \\ &= a^2 + b^2 + c^2\mathrm{Var}(TV^*) + d^2 + 2ab\rho + 2(a+b)c\mathrm{Cov}(T,TV^*), \end{aligned} \tag{6}$$

where in the second step we use that $\mathrm{Cov}(T,TV^*) = \mathrm{Cov}(V,TV^*)$ because of symmetry arguments. To proceed further, we need to establish both $\mathrm{Var}(TV^*)$ and $\mathrm{Cov}(T,TV^*)$.

That $\mathrm{Var}(TV^*) = \mathrm{Var}(T \times V) = 1 + \rho^2$ follows immediately from Bohrnstedt & Goldberger (1969, Eq. (6)).

$$\begin{aligned} \mathrm{Var}(T \times V) &= \mathrm{E}^2(T)\mathrm{Var}(V) + \mathrm{E}^2(V)\mathrm{Var}(T) + 2\mathrm{E}(T)\mathrm{E}(V)\mathrm{Cov}(T,V) + \\ &\quad \mathrm{Var}(T)\mathrm{Var}(V) + \mathrm{Cov}^2(T,V) \\ &= 0 + 0 + 0 + 1 \times 1 + \rho^2. \end{aligned} \tag{7}$$

We establish $\mathrm{Cov}(T,TV^*) = 0$ using Bohrnstedt & Goldberger (1969, Eq. (13)) and supplementary variable $U \sim N(1,0)$ (thus, $U \equiv 1$):

$$\begin{aligned} \mathrm{Cov}(T,TV^*) &= \mathrm{Cov}(T \times V, U \times V) \\ &= \mathrm{Cov}(T,U)\mathrm{Cov}(T,V) + \mathrm{Cov}(T,T)\mathrm{Cov}(V,U) \\ &= 0 \times \rho + 1 \times 0 = 0. \end{aligned} \tag{8}$$

That the covariances of $T$ and $V$ with $U$ are zero is obvious: $U$ does not vary, thus also not covary.

Plugging in (7) and (8) into (6), we obtain

$$\mathrm{Var}(X) = a^2 + b^2 + c^2(1+\rho^2) + d^2 + 2ab\rho.$$

The last column of Table 1 is computed with this formula.

## 2 SPECIFICATION OF PRIOR INFORMATION

In this appendix, we outline how the prior distributions behind the model are specified. To stay in line with the main part of the manuscript we use $N(\mu, \sigma^2)$ as notation for a normal distribution with variance $\sigma^2$. We employ the notation introduced in Equation (2).

The following parts are in common for the models with latent violator, continuous observed violator and dichtomized violator:

$$\begin{aligned}
u &\sim N(0,1) \\
a &\sim N(0,\tau^{-2}) \\
b &\sim \begin{cases} N(0,\tau^{-2}) & \text{for items without uniform bias} \\ N(0.4,\tau^{-2}) & \text{for items with uniform bias} \end{cases} \\
c &\sim \begin{cases} N(0,\tau^{-2}) & \text{for items without nonuniform bias} \\ N(0.4,\tau^{-2}) & \text{for items with nonuniform bias} \end{cases} \\
d &= \tau^{-2} \\
\begin{pmatrix} \mathrm{Var}(T) & \mathrm{Cov}(T,V) \\ \mathrm{Cov}(T,V) & \mathrm{Var}(V) \end{pmatrix} &\sim \mathrm{Wishart}\left( \begin{pmatrix} 1 & \rho \\ \rho & 1 \end{pmatrix}, 4 \right),
\end{aligned}$$

where $\rho$ is either equal to $0$ or $0.5$ (Table 2). Each of the precisions, $\tau^2$ has the $\mathrm{Gamma}(9,4)$ distribution as hyperprior.

In case there is a continuous observed violator (conditions 9–16 in Table 4), we adjust the hyperprior for the precision of the observed violator to $\tau^2 - 1000 \sim \mathrm{Gamma}(4,9)$. This way, we ensure that the precision is extremely large and, thus, the variance is extremely small, making the violator practically static.

In case there is a dichotomized observed violator (conditions 17–24 in Table 4), we adjust value $u$ of the violator to $u \sim N(0, \tau^{-2} = 1/63)$.
